# Planning and designing a self-compassion intervention for family carers of people living with dementia: a person-based and co-design approach

**DOI:** 10.1186/s12877-022-02754-9

**Published:** 2022-01-14

**Authors:** Jenny Murfield, Wendy Moyle, Analise O’Donovan

**Affiliations:** 1 Food & Mood Centre, IMPACT (Institute for Mental and Physical Health and Clinical Translation), School of Medicine, Deakin University, Geelong, Australia; 2 Menzies Health Institute Queensland, Griffith University, Brisbane, Australia; 3 School of Nursing and Midwifery, Griffith University, Brisbane, Australia; 4 Griffith Health Group, Griffith University, Brisbane, Australia

**Keywords:** Alzheimer’s disease, Caregivers, Co-design, Dementia, Intervention development, Mental health, Patient and public involvement, Person-based approach, Qualitative research, Self-compassion

## Abstract

**Background:**

This article describes the research activities undertaken to plan and design a self-compassion intervention for family carers of people living with dementia using a person-based and co-design approach. In providing this example, our aim is two-fold: to highlight the value of using qualitative research and co-design processes within intervention development; and to showcase systematic reporting of an intervention’s early planning and design stages.

**Methods:**

A person-based and co-design approach informed the planning and design of the self-compassion intervention. In Stage 1, qualitative interviews were undertaken with 14 family carers of people living with dementia and 14 professional stakeholders. In Stage 2, intervention guiding principles were developed, psychological theory was incorporated, and six family carers of people living with dementia were engaged as co-designers.

**Results:**

Knowledge generated during intervention planning identified that the intervention should be situated within the concept of compassion more broadly; address misperceptions, fears, blocks, and resistances to self-compassion; and target feelings of shame, guilt, and self-criticism. Subsequent intervention design activities determined that the needs of family carers of people living with dementia were best met by tailoring an existing intervention, namely group-based Compassion-Focused Therapy.

**Conclusions:**

Our systematic approach highlights the value of incorporating in-depth qualitative research and co-design within the intervention development process to prioritise the perspectives and lived experiences of family carers of people living with dementia. The planning and design process outlined provides insight that is applicable to the development of our intervention and complex health interventions within gerontology and beyond.

**Supplementary Information:**

The online version contains supplementary material available at 10.1186/s12877-022-02754-9.

## Introduction

### Family Carers of People Living with Dementia

Providing informal care to a family member living with dementia can be a positive experience, and many carers report feelings of satisfaction and personal reward [1]. However, it can also be challenging, and it is well-established that family carers can be impacted in numerous ways [2]. In terms of psychological and emotional health, studies show that family carers of people living with dementia can experience greater psychological distress than caregivers of other conditions [3, 4]. As many as one in three family carers of people living with dementia experience depression, and one in two report subjective burden [5]. Feelings of guilt and shame are also common within the dementia caregiving role, and heightened levels of both have been associated with the development of depressive symptoms [6, 7].

To help ameliorate some of the described negative impacts, the last few decades have seen an increased focus on the development and testing of different psychosocial interventions to support family carers of people living with dementia [8]. Meta-analyses and reviews have documented encouraging effects for some interventions within clinical trials, and particularly for those that assume a cognitive behavioural approach [8–10]. Most recently, this has included a focus on modern approaches that target mindfulness, acceptance, and compassion, both for the self and others [11, 12].

### Self-Compassion

Self-compassion is variously defined within the literature. In Neff’s [13] conceptualisation, it is understood as treating yourself with care during times of suffering and involves self-kindness rather than self-judgement; common humanity rather than isolation; and mindfulness rather than over-identification. In Gilbert’s [14] understanding, self-compassion is defined within the concept of compassion more broadly, being regarded as part of a three-way ‘flow’ (involving compassion for self, to others, and from others), which involves two aspects: a sensitivity to suffering and a commitment to prevent and/or alleviate that suffering. Drawing on these definitions, research conducted within various clinical and non-clinical populations has found positive links between self-compassion and psychological wellbeing [15] and has shown that self-compassion can be cultivated to improve psychological health [16]. Within dementia family caregiving specifically, similar promising findings have been demonstrated in cross-sectional studies [17, 18], and in a published group intervention study of Compassion-Focused Therapy for couples with a dementia diagnosis [19]. Nevertheless, this understanding is limited to a handful of studies and, as yet there is no self-compassion intervention available for specific use with family carers of people living with dementia.

### Intervention Development

Despite scientific promise, limited numbers of dementia caregiver interventions are translated into real-world use [10, 20], and inadequate reporting of the intervention development process [10, 20] and insufficient understanding of the carers’ needs [e.g., 21] have been implicated. Consistent with health intervention research at large [22, 23], these findings suggest that the development of new and/or alternative interventions to support family carers of people living with dementia should be systematically described and grounded in an in-depth, qualitative understanding of users’ real-life needs and preferences. The Person-Based Approach (PBA) to intervention planning and development [24–26], and Patient and Public Involvement (PPI) through a process of co-design [27–29], offer ideal frameworks to achieve this.

The PBA utilises extensive in-depth qualitative research to situate the intervention in the perspective and lived experience of the people who will use it [24–26]. Although a relatively new approach, it has been successfully used to develop various health interventions for different populations, including family carers [30] and older adults [31, 32]. Offering a flexible approach to intervention development, the PBA involves a qualitative exploration of the key issues, needs, and challenges that the intervention must address, and the formulation of guiding principles that set out the intervention’s key design objectives and their corresponding key features. The approach can be used alongside traditional evidence- and theory-based intervention development frameworks, including the UK Medical Research Council’s (MRC) guidance [33, 34]. It is also advocated for use with methods of PPI, including co-design [35].

Defined as doing research ‘with or by’ public and patients rather than doing it ‘to, about, or for’ them [28, 29], PPI is universally acknowledged as a valuable and important part of the research process that can result in the production of interventions that have greater relevance to everyday practice [36]. Within dementia research, PPI is rapidly increasing [37], and a growing number of intervention development studies are involving family carers and people living with dementia in the planning and design stages as co-designers [37–39]. Nonetheless, the processes by which researchers have undertaken co-design are not well documented, and greater transparency in reporting is needed to facilitate best practices [37].

### The Current Study

Building on the described work, we sought to develop a self-compassion intervention for family carers of people living with dementia. The purpose of this article is to describe the research activities we undertook to plan and design this intervention using a person-based [24–26] and co-design approach [27–29]. By providing this example, we aim to highlight the value of using qualitative research and co-design to prioritise the perspectives and lived experiences of the intervention’s intended target users, and to showcase a systematic approach to the early planning and designing stages within intervention development. To promote rigour in our reporting, we have used the Guidelines for the Reporting of Intervention Development Checklist (GUIDED) [40] and the Guidance for Reporting Involvement of Patients and the Public (GRIPP2-SF) [41] (see Additional file 1).

## Methods

### Intervention Planning and Design Process

The complete intervention development process used evidence-based, theory-based, person-based, and co-design approaches to inform the planning and design of the self-compassion intervention [24–29, 33, 34]. Although not described within this article, we first drew on the ‘development’ phase of the MRC framework for best practice in developing and evaluating complex health interventions [33, 34]. In brief, this involved three evidence- and theory-based activities: (1) literature reviews [12, 42]; (2) conceptual analysis [43]; and (3) cross-sectional survey study [18, 44]. Relevant to this article’s focus, we then chose to supplement the MRC framework with additional qualitative and co-design approaches to ensure that the intervention was grounded in the perspective and lived experience of the intervention’s target users. For this, we first drew on the ‘planning’ and ‘design’ phases of the PBA for developing behavioural interventions [24–26]. We then incorporated the principles of PPI in health and medical research [27] by using a co-design process that broadly aligned with the ‘deciding how to do it’/‘designing and managing’ phase of the research cycle [28, 29].

The person-based and co-design approach reported within this article comprised two research stages that focused on (1) intervention planning, and (2) intervention design. In Stage 1, we undertook qualitative interviews with family carers of people living with dementia and professional stakeholders to explore perceptions, barriers, facilitators, and contextual issues pertinent to planning the intervention. In Stage 2, we undertook a co-design process that saw us engage a small group of family carers of people living with dementia to assist in decision-making about the intervention’s design, including the development of guiding principles, the incorporation of psychological theory, and the creation of fictional scenarios and personas.

 We received ethical approval for the study from Griffith University (Ref: 2019/481), and we obtained written informed consent and verbal assent from qualitative participants and co-design group members.

### Stage 1 Intervention Planning: Qualitative Interviews

To explore perspectives about the proposed intervention and identify any potential barriers, facilitators, and contextual issues relevant to its design, we conducted semi-structured interviews with 14 family carers of people living with dementia (aged ≥18 years and self-identifying as a family carer of a person living with dementia) and 14 professional stakeholders (academic clinicians with expertise in ageing/dementia, family caregiving, and/or compassion, and carer support professionals). We recruited family carers of people living with dementia from Australia using convenience sampling. This involved participants voluntarily responding to social media posts, promotions in carer organisations’ electronic newsletters, and in-person talks at carer support groups (within 60kms of Brisbane, Queensland). We recruited professional stakeholders using purposive sampling. This involved the lead author emailing professional contacts with known relevant expertise from Australia and the UK.

Between September and December 2019, the lead author conducted individual, one-off, verbal, semi-structured interviews with participants. Interviews were conducted either by telephone (*n* = 16), via videoconferencing (*n* = 9), or in-person (*n* = 3) and averaged 30 minutes in duration (range 15 – 62 min). We used three interview schedules that we tailored slightly to accommodate participant groups’ differing contexts. However, across all interviews, we asked participants a core set of questions about their understanding of self-compassion as a concept; their thoughts about a self-compassion intervention for family carers of people living with dementia; and the things that they thought might help or hinder intervention implementation, including methods and modes of delivery. We digitally audio-recorded interviews and transcribed them verbatim, and we made analytical field notes. To analyse the data, we used a recursive process of inductive, reflexive thematic analysis [45, 46]. This involved: (1) repeatedly listening to and reading the transcripts to become familiar with the data; (2) line-by-line coding on hard-copies of transcripts to generate initial codes; (3) grouping codes with a shared meaning to generate initial themes and subthemes; (4) reviewing the developing themes and subthemes for meaning against the study’s aim; and (5) defining the developed themes using supporting quotations from the data. The lead author undertook this analysis and met with the authorship team multiple times to discuss the developing coding frame and to reflect on their interpretative judgement of the themes identified in relation to the aims of the analysis [47]. Consistent with the assumptions of reflexive thematic analysis [48], the total number of interviews we conducted was pragmatically determined, seeing the interviews individually and collectively reviewed for their adequacy (i.e., richness and complexity) to meet the study’s aims.

### Stage 2 Intervention Design: Co-Design Group

To ensure that the design of the proposed self-compassion intervention was best suited to the real-life needs and preferences of its intended target users, we engaged six family carers of people living with dementia to work alongside the first author as co-designers. Using convenience sampling, we recruited co-design members (≥18 years and self-identifying as a family carer of a person living with dementia) from Australia. This involved the lead author posting advertisements on social media and emailing/writing to known family carers of people living with dementia. We did not require co-design members to have any experience or training in research methods; however, all had participated in at least one research project previously, independent of this study and the authors.

Between October and November 2020, all co-design members took part in four, 90-minute sessions that were conducted weekly. Five of the co-design members took part as a group and participated in the sessions online using Microsoft Teams videoconferencing. One co-design member opted to participate individually, as based on their preference to engage independently rather than in a group. For each week’s session, this involved them watching a pre-recorded video that mirrored the same content as the online session, and then participating in a follow-up telephone conversation. In keeping with recommendations for PPI within dementia care research [39], we financially reimbursed all co-design members for their involvement (AUD132 per session).

The lead author was trained in conducting qualitative and group-based discussions with this population and facilitated all sessions. Although we used a session agenda, we were flexible and adopted an iterative approach by covering content in sessions as necessary. Sessions were video and/or digitally audio-recorded and collectively covered the following content: discussion, feedback, and refinement of the intervention’s formulated guiding principles; discussion and feedback on the proposed structure and broad content of the proposed intervention; and the creation of fictional caregiver scenarios and personas for use in the intervention.

## Results

### Stage 1 Intervention Planning: Qualitative Interviews

From the 28 interviews we conducted exploring the potential barriers, facilitators, and contextual issues pertinent to the intervention’s design, we inductively identified five relevant themes and 12 sub-themes. Given that the purpose of this article is to describe the intervention development process, we have chosen to present participant characteristics (Table 1) and a selection of de-identified participant quotations to support the final themes (Table 2) within a tabular form, rather than including these data within the text directly.

**Table 1:** Characteristics of qualitative interview participants and co-design group members

Characteristic	Descriptive statistics
**STAGE 1: QUALITATIVE INTERVIEWS**	
Family carers of people living with dementia (*n* = 14)	
Age (years)^a^	62.5 (14.4)
Identifying gender (Female: Male)^b^	11 (78.6): 3 (21.4)
Country of residence (Australia)^b^	14 (100)
In employment (Yes: No)^b^	4 (28.6): 10 (71.4)
Relationship to care recipient (Partner: Offspring)^b^	7 (50): 7 (50)
Length of time caring for care recipient (years)^a^	6.5 (2.9)
Age of care recipient (years)^a^	78.6 (10.4)
Identifying gender of care recipient (Female: Male)^b^	9 (64.3): 5 (35.7)
Care recipient’s type of dementia^b^	
Alzheimer’s disease	7 (50)
Unspecified	4 (28.6)
Frontotemporal	1 (7.1)
Lewy-body	1 (7.1)
Vascular	1 (7.1)
Professional stakeholders (*n* = 14)	
Academic clinicians with expertise in ageing & dementia (*n* = 5)	
Identifying gender (Female: Male)^b^	4 (80): 1 (20)
Country of residence (Australia)^b^	5 (100)
Role^b^	
Clinical psychologist	1 (20)
Mental health nurse	1 (20)
Occupational therapist	1 (20)
Old age psychiatrist	1 (20)
Social gerontologist	1 (20)
Academic clinicians with expertise in compassion (*n* = 4)	
Identifying gender (Female: Male)^b^	2 (50): 2 (50)
Country of residence (Australia: UK)^b^	1 (25): 3 (75)
Role^b^	
Clinical psychologist	3 (75)
Counsellor	1 (25)
Carer support professionals (*n* = 5)	
Identifying gender (Female: Male)^b^	4 (80): 1 (20)
Country of residence (Australia)^b^	5 (100)
Role^b^	
Counsellor	2 (40)
Senior management (education, training, & improvement)	2 (40)
Educator	1 (20)
**STAGE 2: CO-DESIGN GROUP**	
Family carers of people living with dementia (*n* = 6)	
Age (years)^a^	61 (8.0)
Identifying gender (Female: Male)^b^	5 (83.3): 1 (16.7)
Country of residence (Australia)^b^	6 (100)
In employment (Yes: No)^b^	3 (50): 3 (50)
Relationship to care recipient (Partner: Offspring)^b^	1 (16.7): 5 (83.3)
Length of time caring for care recipient (years)^a^	5.8 (2.8)
Age of care recipient (years)^a^	86 (9.2)
Identifying gender of care recipient (Female: Male)^b^	4 (66.7): 2 (33.3)
Care recipient’s type of dementia (Alzheimer’s disease: Mixed)^b^	4 (66.7): 2 (33.3)

*Note.* ^a^continuous variables are reported as *M* (*SD*); ^b^categorical variables are reported as *n* (%)

**Table 2:** Themes identified in Stage1 with selected supporting quotations from qualitative interviews

Theme	Family carers of people living with dementia		Professional stakeholders
**Understanding of self-compassion**			
*Unfamiliar concept and word*	‘That’s the thing that had me a little bit confused. Self-compassion? I’m compassionate about my wife. I’m compassionate, but self-compassion? That’s the one that beat me.’ (#5 male carer) ‘You need something that is not quite esoterical and not so academic because there’s an awful lot of the population that wouldn’t understand that concept.’ (#14 female)		‘Use language that will make sense to most people, and I don’t know if people recognise the term self-compassion.’ (#1 female, AC/(A/D), social gerontologist) ‘Well, I had actually never heard the term [self-compassion] before, so when my manager sent me this email, I sort of went, oh, what does that mean?’ (#6 female, CSP, educator)
*Related to compassion more broadly*	‘I reckon in looking after Mum I am looking after myself because I want to do it. Now, to me, that’s nourishing me because I can see the benefit.’ (#2 female) ‘I get care workers and have strangers living in my house from overseas because that helps take care of things around the house that I can or can’t do. And allowing people into your life that do want to be there…to me, that’s a form of compassion.’ (#8 female)		‘I wouldn’t make necessarily a big distinction between self-compassion and compassion in general. I think where people often get stuck is that they assume that compassion for someone else is the only kind of compassion and, hence, why people put the self-compassion on there as well…You know, it’s something to say that, if you want the help, you can get it, and you can get that help by actually looking at where you stand with yourself. Not doing more for the person you care for but being kinder to yourself will lead to a greater capacity of being kind to others…I think it’s embodying the practice. If you want to be a compassionate individual, you can’t do that while taking big chunks out of yourself and berating yourself.’ (#8 male, CSP, counsellor)
**Perceptions of self-compassion**			
*Positive perceptions, but with some qualifiers*	‘I think that the thing that compassion does give you, is it gives you advocacy for your own voice to finally be heard … But I don’t think self-compassion should ever include the excuse that the behaviour is allowed to continue. I think you need self-compassion to understand that they [the person with dementia] don’t know what they’re doing. They are vulnerable and that they are totally reliant.’ (#8 female)		‘I think the ability to tolerate negative affect and to appreciate that some things can’t be easily changed and that tolerating, you know, an unpleasant situation is sometimes all we can do and that continuing to rail against it is often unproductive. So, I think those lessons are probably worth teaching. But not just by themselves…sometimes it’s reasonable to not continue caring or to engage other people to assist you rather than to tolerate an intolerable situation.’ (#2 male, AC(A/D), old age psychiatrist)
*Negative perceptions (resistance)*	‘I don’t know whether it’s a bit of self-indulgence…I saw it as, oh no, is this one of those ones where you’re important, well you’re at the centre, so you need to look after yourself.’ (#2 female) ‘Often when you’re thinking about self-compassion you feel that it’s purposefully selfish to be considering that.’ (#3 female)		‘Compassion, they have compassion for others, but self-compassion seemed to be interpreted as self-pity.’ (#7 female, CSP, counsellor) ‘I think there is that sense of self-indulgence… people I’ve spoken to, the carers and things like that, where they’re probably so busy and so focused on their loved one that it would almost seem self-indulgent to focus on themselves even though we know how important that is.’ (#1 female, AC/(A/D), social gerontologist)
*Family carer background characteristics*	‘I think my generation have always been brought up to show that we’re tough and that we’re the ones running the families and that everyone comes back to us and listens to and takes advice from.’ (#11 female carer) ‘And a lot of it is about what they’re brought up with too…like with cultural expectations or parents who sort of had their parents deal with the grandparents.’ (#14 female)		‘Part of the issue is the expectations, especially those familial expectations…And so often generational expectations, gender expectations come into play and so if you say…you could treat yourself with some more care you could be more compassionate towards yourself and that will help you, that might lead to, well, I don’t need to or that was never taught to me…’ (#8 male, CSP, counsellor) ‘And even to think about myself is selfish, and a lot of folk would say I was raised at a time when you didn’t do that, you absolutely thought of other people.’ (#14 male, AC(C), clinical psychologist)
**Realities of cultivating self-compassion**			
*Demands of the caregiving role*	‘I think any caring role has to be very, very draining and doesn’t leave a lot of space for self-compassion. And there’s a lot of rhetoric around that and people constantly provide the advice of, oh do take care of yourself. But that is so bloody difficult. It really is. There’s not a lot of time, there’s not a lot of space and there’s very little energy for it.’ (#6 female) ‘[The] time available for carers to come to something like that can fluctuate, can vary, so, because the focus is on the other person they may not, you know, create time for themselves.’ (#9 female)		‘Whether they’ve got time to take care of themselves is another thing, you know. It has to be a priority for them because they find it really hard to be able to do that.’ (#10 female, AC(A/D), mental health nurse) ‘A lot of them [carers] use words like survival and I think that self-compassion is really higher order, and it does require their time and commitment, and I just don’t know if a lot of carers have gotten there yet.’ (#6 female, CSP, educator)
*Outward focus*	‘I think when you’re caring for somebody with dementia or Alzheimer’s, your focus is on that person in the first instance. And so, thinking about oneself is not the first thing that comes to mind…So it’s external-facing as opposed to looking within.’ (#9 female) ‘You feel like you’re always needing to put the other person first because their needs, whether that be mental or emotional, are always feel greater than your own.’ (#3 female)		‘…self-compassion just doesn’t even come into their head space or just isn’t a thing that they can think of doing…It strikes me that they could be quite outward focused rather than inwardly focused.’ (#5 female AC/(A/D), clinical psychologist) ‘I think, carers are often…very much in automatic pilot. A lot of people [carers] talk about being in a routine, which then stops them from looking beyond that. Like, stopping to look at what it’s like for them.’ (#9 female, CSP, senior management)
**Fears of self-compassion**			
*Capacity and willingness to open-up*	‘The reason I’m restrained now is because of Mum. We don’t raise our voices, we just don’t. We don’t have any emotions floating around from one end of the house to the other …because when you’ve got dementia, if something’s not right, that can go on for hours and hours, and that’s very damaging. So, I’ve taught myself how to hold onto all that stuff, but I suspect my volcano will explode in the future’. (#2 female)		‘…by self-reflecting and realizing, there’s the potential for, oh my god, this is how I’m feeling…Some people, sort of, are very, very wary of allowing themselves to open up to that…’ (#9 female, CSP, senior management) ‘You may just find people [carers] come into that space saying, look, I really want this, but I can’t. I just can’t do what you want me to do because it’s such a threat.’ (#8 male, CSP, counsellor)
*Shame, guilt, and self-criticism*	‘People are telling me I’m doing a really good job and I agree. So, there’s that. But, you know, there’s times when I just feel worthless, so it’s a roller coaster.’ (#4 female) ‘Being a carer, you think that you’re suffering, alone, which increases your stress levels and then you sort of self-criticise yourself.’ (#11 female)		‘They often feel guilty about taking a break or they often feel guilty if they have negative thoughts about the person they’re caring for, even though they are very common and probably quite normal.’ (#2 male, AC(A/D), old age psychiatrist) ‘I think it’s hard to talk about compassion without talking about shame…[carers can feel] lesser than the person that’s the priority and that can begin to erode their sense of self…. how do I be compassionate towards myself if the fundamental core of me, I feel ashamed about…We need to look at shame and of the ways out of shame is to look at compassion.’ (#8 male, CSP, counsellor)
**Supporting attendance and implementation**			
*Importance of pitch*	‘It’s gonna be tricky to sell…I think the problems that you’re going to have is that, especially with long-term carers, is that we’ve got a bit jaded because we go to these things and it’s always, it always becomes academic…So it really needs to be promoted as a, as something for you, this is what you can do.’ (#7 female)		‘Convincing people that this is a good idea. I think that’s gonna be the biggest challenge.’ (#10 female, AC(A/D), mental health nurse) ‘The problem is, I think, trying to pitch it in a way that, we know you’re [carers] busy, we know you’re overwhelmed, we still think you should come along to do whatever.’ (#1 female, AC/(A/D), social gerontologist)
*Flexible, responsive, practical delivery*	‘Until they can accommodate our loved one, it’s actually creating a burden, which is why a lot of us are doing the courses online… Online is one way, in terms of it being cost-effective.’ (#8 female) ‘As long as there’s flexibility built into it and, of course, best practice would be that the carer comes for those eight weeks for one or two hours a week. But I think, if it’s communicated in a way that if you were to, sort of, dip in and out, then that’s not the end of the world and you’d still get benefit from it. So, if you were to miss a week then you wouldn’t have to completely withdraw.’ (#3 female)		‘So, it has to speak to people about their daily life and experience…I think there’s a lot of value in something that’s really punchy and skill-based so that people can do something with the information right away and the time limited nature of it…people are there to get as much out of it as they can, and they want something useful that they can immediately apply to their lives.’ (#6 female, CSP, educator) ‘…if you are a carer then sometimes you can’t get out of the house or you can make an appointment six weeks ahead…So, I think a model that’s really flexible and responsive’. (#1 female, AC(A/D) social gerontologist)
*Skilled facilitation*	‘I’ve sat in a lot of them, carers’ forums and things like that, and it’s always difficult to stop the conversation or guide the conversation away from your woes as a carer, or your concerns, and steer back to what the focus is.’ (#3 female) ‘What I met at that meeting [a carer support group], I can’t see that it served any purpose whatsoever. It seemed to be a competition in who had had it worse. To me it was badly regulated.’ (#2 female)		It needs to be well facilitated by people who can sit with what comes up in the room. They have to feel comfortable in allowing people to go where they want to in the session, you know, without it being a counselling session.’ (#7, female, CSP, counsellor)

*Note.* AC(A/D) = academic clinician (ageing/dementia); AC(C) = academic clinician (compassion); CSP = carer support professional

#### Understanding of Self-Compassion

Self-compassion was a largely unknown concept to participants. Some family carers of people living with dementia had never heard of self-compassion before, and a minority of professional stakeholders with expertise in ageing and dementia were unfamiliar with the concept. When describing what they understood self-compassion to be, it was common for participants to situate self-compassion within the concept of compassion as it related to others more broadly (i.e., giving and receiving). Specifically, some of the family carers of people living with dementia we interviewed had never considered giving compassion to themselves and only understood compassion as relating to caring for another person. Other comments from participants reflected the bidirectional relationships between self-compassion and compassion for others (i.e., the importance of caring for the self in order to care well for another/caring for another as a way of caring for the self), as well as the relationship between self-compassion and being open to receiving compassion from others (i.e., accepting and bringing in outside help).

#### Perceptions of Self-Compassion

In the main, participants perceived self-compassion positively, describing potential benefits for carers in helping with healthy emotion regulation; reducing self-criticism and feelings of guilt; enhancing resilience; enabling self-advocacy; and enhancing carers’ ability to self-evaluate. That said, some participants also added important qualifiers: self-compassion should not be used to either excuse or allow psychological or physical abuse within a caregiving relationship, or for a family carer to remain in an acutely stressful situation.

One family carer of a person living with dementia was overtly critical of self-compassion, seeing it as self-indulgent and focused solely and unnecessarily on the self. Although personally supportive of the concept, other family carers of people living with dementia also commented that self-compassion could be interpreted as selfish, self-indulgent, and related to the self-care rhetoric commonly heard in dementia carer support services and the wider general discourse. These sentiments were echoed by professional stakeholders, whereby it was considered likely that some family carers of people living with dementia may be resistant to self-compassion due to associations with self-indulgence, self-pity, and weakness. Family carers’ individual background characteristics, such as stoicism and gender-, role-, and cultural-based expectations, were considered possible influences on these perceptions of self-compassion.

#### Realities of Cultivating Self-Compassion

The constant and demanding nature of being a family carer of a person living with dementia was highlighted by participants as one of the main blocks to carers attending a self-compassion intervention and embedding the practices within everyday life. There was a perception that many family carers were often overwhelmed and, therefore, may either not be open to self-compassion or would view the required activities and practices as another stressor.

Many participants considered family carers to be outward focused in their efforts (i.e., placing the needs of the care recipient first) and, as such, spent little time on inward work on the self. Professional stakeholders also thought that because family carers did not routinely prioritise themselves, self-compassion was not something that family carers would either think of or potentially consider possible for themselves.

#### Fears of Self-Compassion

 Although the opportunity for self-reflection and the cultivation of self-compassion was seen as a positive thing for emotional health, participants also recognised that this could be challenging for many carers. There was a perception that family carers of people living with dementia often avoided emotional reflection to enable them to continue in the caregiving role and through fear of emotional breakdown. For some family carers of people living with dementia, it was thought that emotional reflection could lead to negative thoughts about their situation and, ultimately, the person they were caring for, which could then lead to feelings of guilt and shame. It was also thought that family carers of people living with dementia could be particularly self-critical and that emotional reflection may exacerbate these feelings (i.e., highlighting that they were not as compassionate to themselves as they should be).

#### Supporting Attendance and Implementation

 Participants commented that the intervention’s success would likely depend on how it was pitched. Although some participants thought it helpful to demonstrate the potential benefits of the intervention for both the carer and the care recipient (i.e., will support you to be a better carer), there was a more common view that to ensure clarity about the intervention’s focus, it was important to be explicit that the focus was on the carer personally.

 Due to the nature of the caring role, participants stressed that the intervention needed to be as flexible, responsive, and practical as possible. Psychoeducation and practical skills-based learning that was relevant and able to be incorporated into daily life were highlighted. Additionally, with carers limited in their ability to attend sessions due to their role, the mode of delivery was raised. The potential for online delivery (rather than face-to-face) was the main suggestion, although some stakeholders highlighted reduced efficacy and technical issues as considerations, as well as potential issues with recruitment.

 Participants acknowledged the importance of having a trained and skilled facilitator to run the intervention group. Many commented on the sometimes-judgmental nature of carers with each other, which, if not facilitated well in group work, could be unproductive. Professional stakeholders also specifically commented on the need for the intervention to be led by a trained mental health professional.

Most participants highlighted the need to consider alternative care provision for the care recipient, particularly if the intervention required in-person attendance. There was a prevailing view against undertaking dyadic group work (i.e., carers and care recipient in the group together), as this may inhibit carers from talking about their feelings and situation. However, one stakeholder had undertaken dyadic work with family carers of people living with dementia within a similar area, and this had been successful.

### Stage 2 Intervention Design: Co-Design Group

Drawing on the themes generated from the in-depth qualitative interviews conducted in Stage 1 and the findings from our earlier evidence- and theory-based activities (see [12, 18, 42, 43]), we developed draft guiding principles and drew on psychological theory to inform the proposed design of the intervention. We then convened a co-design group and presented both aspects to them for discussion, feedback, and/or refinement. Alongside this, the co-design group also assisted in creating caregiver scenarios and personas for use in the intervention. Table 1 provides the background characteristics of the six co-design group members.

#### Guiding Principles

Our intervention’s guiding features focused primarily on the importance of addressing potential misperceptions, fears, blocks, and resistances to self-compassion and how attendance could be best supported through consideration of issues related to intervention delivery. Co-design group members agreed with the content of the guiding principles; however, they felt that the language used to define the key issues, design objectives, and features was unnecessarily complex and not easy for them to understand. Therefore, to ensure that the principles of PPI were upheld throughout the process of the intervention’s development (i.e., challenging potential power imbalances between the researchers and co-design members) the language used in the guiding principles was simplified. This was an important step in designing the intervention and ensured that co-design members were able to contribute equally and with confidence to the process. Table 3 provides an overview of the finalised guiding principles, which were iteratively developed and agreed upon in consultation with the co-design group.

**Table 3:** Guiding principles for the self-compassion intervention

Key issue	Design objective	Key intervention features
Self-compassion is an unfamiliar term to many family carers of people living with dementia and it is often understood in relation to the concept of compassion more broadly	• To provide family carers of people living with dementia with a better understanding of what self-compassion is and how it relates to the concept of compassion more broadly (i.e., compassion for self, to others, from others)	¬ Provide a clear definition of compassion and define self-compassion within the concept of compassion more broadly (i.e., compassion for self, to others, from others) ¬ Differentiate compassion with similar concepts/feelings (i.e., empathy, sympathy, apathy, self-esteem) ¬ Highlight evidence-base supporting the benefits of compassion ¬ Use clear, simple, non-academic language
Self-compassion is generally perceived as a positive concept; however, some family carers of people living with dementia will hold negative perceptions and may be initially resistant to the idea of self-compassion	• To address and clarify family carers’ misperceptions of self-compassion and facilitate understanding about why family carers of people living with dementia may feel resistant to the idea of being self-compassionate	¬ Address and clarify family carers’ potential discomfort to the idea of self-compassion (e.g., societal expectations, cultural influences etc.). ¬ Frame self-compassion as beneficial to the caregiving role, but not as a panacea/fix-all ¬ Use examples and metaphors that relate to the caregiving role
Family carers’ ability to cultivate self-compassion may be inhibited by the demands and nature of the caregiving role	• To highlight the practical blocks to cultivating self-compassion in the caregiving role and focus on using exercises and practices that can be easily incorporated into family carers’ daily lives	¬ Validate the 24/7 demands of the caregiving role that can make cultivating self-compassion hard ¬ Highlight the relationship between the three aspects of compassion (i.e., compassion for self, to others, from others) and how the outward focus of the role (compassion for others) might influence their ability to be self-compassionate ¬ Use exercises and practices that can be easily incorporated into family carers’ daily lives/routines
Some family carers of people living with dementia may have limited capacity and willingness to engage in self-compassion for fear of emotional reflection that could lead to feelings of shame, guilt, and self-criticism	• To address and validate family carers’ fears of self-compassion and facilitate understanding about how self-compassion may help with feelings of shame, guilt, and self-criticism	¬ Validate the need to ‘keep going’ as a family carer and the fear of emotional reflection, but highlight the costs of not attending to the emotional needs of the self ¬ Deliver psychoeducation on how self-compassion can help with feelings of shame, guilt, and self-criticism
Implementation of the intervention may be enhanced by considering issues related to pitch, mode of delivery, facilitation, and alternative care support arrangements	• To address practical issues that could inhibit family carers’ attendance and consider factors that may hinder implementation	¬ Promote the intervention using simple language, emphasising an explicit focus on the carer but with benefits to the caring role more broadly ¬ Offer different/mixed modes of delivery to support carer learning and attendance needs ¬ Throughout the intervention address differences in understandings, perceptions, and experiences of compassion depending on the family carers’ background characteristics (i.e., relationship; family dynamics; age; culture; gender etc.) ¬ Emphasise delivery by trained facilitators ¬ Help/sign-post family carers to access care support arrangements (both formal and informal) to enable attendance

#### Incorporating Psychological Theory

The knowledge collectively generated during intervention planning inductively identified that it was important for the self-compassion intervention to be presented to family carers of people living with dementia in a way that situated it within the concept of compassion more broadly; addressed misperceptions, fears, blocks, and resistances to self-compassion; and targeted feelings of shame, guilt, and self-criticism. Drawing on the psychological literature, we therefore determined that, rather than developing a novel self-compassion intervention, the needs of family carers of people living with dementia could be best met by tailoring an existing approach: group-based Compassion-Focused Therapy (CFT) [14].

CFT is an integrative approach that aims to develop compassion (both for the self and others) to improve emotional wellbeing by targeting shame and self-criticism [14]. It defines compassion as a motivation that can be directed towards others, from others, or towards the self (self-compassion), and it works explicitly with fears, blocks, and resistances to compassion [14]. Delivered by trained facilitators, the approach has traditionally focused on the dynamic therapeutic process and therefore has not been manualised. However, a standardised, 12-module group CFT manual is under development (Gilbert, Kirby and Petrocchi) and, although not publicly available, a few studies have been given access and successfully tailored the manual for use with specific populations [49, 50].

We provided co-design group members with a brief theoretical introduction to CFT and an overview of the 12-module manual, as used and described in the study by Carter et al. [50]. We then asked the group for their thoughts on how CFT aligned with the guiding principles and for their general perceptions of CFT as an intervention for use with family carers of people living with dementia. All co-design members agreed that there was a strong alignment of CFT with the formulated guiding principles and fully supported tailoring the group CFT for our population of interest.

#### Creation of Caregiver Scenarios and Personas

In line with the PBA’s recommendation to undertake user-centred designed [26], we asked co-design members to create scenarios and personas for use within the intervention that were about any aspect of the guiding principles (i.e., perceptions, fears, blocks, resistances etc.). We presented a working example, and each co-design member worked individually to draft their scenarios. Collectively, fourteen fictional scenarios and personas were created, and these can be used within the developed intervention to help ground the content to the experiences of family carers of people living with dementia. For illustrative purposes, two of the created scenarios are provided below:

The first introduction to self-compassion was during a stressful, demanding and exhausting time in my life and I heard the term and it just sounded so reminiscent of the popular positive pop psychology books, workshops etc. that sound great, take your money and you are no better off.

George was reluctant to join a group to develop his feelings of compassion. He hadn’t had a good experience in any of the other carer groups he’d joined, and he felt uneasy about revealing too much of himself to others. He was filled with so much grief at watching the person he loved most in the world fade away. He felt a shadow of the person he was before. He knew the caregiver journey had changed him but how could he explain that within a group where others might judge him?

## Discussion

Within this article, we have described the research activities undertaken in planning and designing a self-compassion intervention for family carers of people living with dementia that used a person-based and co-design approach. By systematically reporting the process we took, we offer insight into planning and designing an intervention that is applicable to both the development of our intervention and the development of complex health interventions generally. Additionally, we also show the value of incorporating in-depth qualitative research and co-design within the intervention development process, with our findings determining that the needs of our population were best met by tailoring an existing intervention rather than developing a novel intervention.

 The qualitative research and co-design group we undertook generated knowledge that ensured that the intervention was planned and designed with the needs and preferences of family carers of people living with dementia at its core. Although some of these issues were identified in our earlier evidence- and theory-based activities [12, 18, 42–44], without this in-depth qualitative and co-design work, we would not have understood their centrality to the acceptability of the intervention for our population. Specifically, during the initial planning stage, we found that dementia family carers’ perceptions of self-compassion aligned with Gilbert’s [14] conceptualisation, seeing it situated within the concept of compassion as it relates to self and others. We also found that family carers of people living with dementia had several misperceptions about self-compassion, as well as some fears, blocks, and resistances that could lead to enhanced feelings of shame, guilt, and self-criticism. These findings align with research conducted with various populations (e.g., [51–54]), including family carers of people living with dementia (e.g., [6, 7, 55, 56]), and thus supports their importance within our intervention’s design. Alongside this, we also found that acceptability of, and likely engagement with, the intervention was influenced by implementation issues, including pitch, method of delivery, facilitation, and alternative care support arrangements. Similar issues were raised as important factors during the recruitment and retention of dementia family carers to a mindfulness-based intervention [57], and therefore indicates their need for consideration within comparable interventions such as ours.

During the design stage, we then used the described findings to determine the intervention’s key content and key features to maximise user acceptability and engagement, which involved the formulation of guiding principles, incorporation of psychological theory, and PPI through a process of co-design. In doing so, we identified that the needs of family carers of people living with dementia were keenly aligned with CFT [14] and that, rather than developing a novel intervention, the soon-to-be-published group manual (Gilbert, Kirby and Petrocchi) affords an opportunity to tailor CFT to our population’s needs. Specifically, CFT conceptualises self-compassion within compassion more generally; is delivered by a trained facilitator; targets shame and self-criticism; and works explicitly with fears, blocks, and resistances to self-compassion. To our knowledge, only one small-scale study has been published reporting the outcomes of group-based CFT with family carers of people living with dementia [19]. Importantly, however, this intervention differs from ours, as it was not manualised and was dyadic in its delivery (i.e., involving people living with dementia and their spouses).

### Limitations

First, although our approach to planning and designing the intervention was systematic, it could be regarded as lengthy and limited to situations where resources of both time and funds are adequate. However, the PBA is non-prescriptive in its application [24–26], and researchers can adapt the research activities to meet their project-specific needs. Second, although we sampled interview participants and co-design members with different characteristics, the majority of our participants were Australian women providing care to either a parent or partner living with dementia. As such, the generalisability of our findings beyond this group is not guaranteed. Third, the lead author conducted all interviews and co-design sessions as part of their doctoral research program. This may have introduced social desirability, with potentially more favourable opinions and preferences expressed to support the lead author. Fourth, due to COVID-19, all PPI was conducted via remote co-design sessions using either videoconferencing or telephone. Although co-design group members informally expressed satisfaction with their involvement in this way, we acknowledge that in-person sessions may have generated a richer experience and data [58].

### Future Research

Building on the knowledge produced during this study, the proposed intervention can progress to the next stages of development and feasibility testing by continuing to use an evidence-based, theory-based, person-based, and co-design approach [24–29, 33, 34]. Next steps will see the guiding principles used to tailor the CFT group manual (once publicly available) for family carers of people living with dementia; all intervention-related documents developed (i.e., information sheets, advertisements etc.); and the intervention’s mode of delivery finalised. After this, the acceptability of the intervention’s components can be tested using think-aloud techniques with family carers of people living with dementia, professional stakeholders, and co-design group members. To ensure that the intervention is optimised to the needs of our population, this may see iterative changes made to the intervention up until it is deemed ready for acceptability and feasibility evaluation.

## Conclusions

With a recognised evidence-to-practice gap for interventions within gerontology, new and alternative approaches to supporting family carers of people living with dementia should be developed in ways that are systematic and have the needs and preferences of intended users at the centre. This article provides an example of how in-depth qualitative research and co-design processes were systematically used to plan and design a self-compassion intervention for family carers of older adults. The approach highlights the potential of using the PBA and co-design in intervention development, with research activities determining that the needs of our population were optimally met by tailoring an existing intervention. Further, the systematic reporting of the planning and design process offers useful insight that is applicable to both our intervention and those interested in developing complex health interventions more broadly.

## Supplementary Information

**Additional file 1.** GUIDED and GRIPP2-SF Reporting Guideline Checklists. Completed reporting guidelines checklists for GUIDED (guidelines for reporting intervention development studies) and GRIPP2-SF (guidelines for reporting involvement of patients and the public- short form)

## Data Availability

The datasets generated and/or analysed in this study cannot be made publicly available as participants did not provide consent for their data to be shared. Further details about the data and ethical conditions for access are available from the corresponding author.

## Abbreviations

CFT: Compassion-Focused Therapy
GUIDED: Guidelines for the Reporting of Intervention Development Checklist
GRIPP2-SF: Guidance for Reporting Involvement of Patients and the Public
MRC: Medical Research Council
PBA: Person-Based Approach
PPI: Patient and Public Involvement

## References

[1] Yu DSF, Cheng ST, Wang J (2018). Unravelling positive aspects of caregiving in dementia: An integrative review of research literature. Int J Nurs Stud.

[2] Merrilees J (2016). The impact of dementia on family caregivers: What is research teaching us?. Curr Neurol Neurosci Rep.

[3] Pinquart M, Sorensen S (2003). Differences between caregivers and noncaregivers in psychological health and physical health: A meta-analysis. Psychol Aging.

[4] Teahan Á, Lafferty A, Cullinan J, Fealy G, O’Shea E. An analysis of carer burden among family carers of people with and without dementia in Ireland. Int Psychogeriatr. 2020:1–12 10.1017/S1041610220000769. 10.1017/S1041610220000769 32484125

[5] Collins RN, Kishita N (2020). Prevalence of depression and burden among informal care-givers of people with dementia: A meta-analysis. Ageing Soc.

[6] Losada A, Marquez-Gonzalez M, Vara-Garcia C, Gallego-Alberto L, Romero-Moreno R, Pillemer K (2018). Ambivalence and guilt feelings: Two relevant variables for understanding caregivers’ depressive symptomatology. Clin Psychol Psychother.

[7] Martin Y, Gilbert P, McEwan K, Irons C (2006). The relation of entrapment, shame and guilt to depression, in carers of people with dementia. Aging Ment Health.

[8] Teahan Á, Lafferty A, McAuliffe E, Phelan A, O’Sullivan L, O’Shea D (2020). Psychosocial interventions for family carers of people with dementia: A systematic review and meta-analysis. J Aging Health.

[9] Kishita N, Hammond L, Dietrich CM, Mioshi E (2018). Which interventions work for dementia family carers? An updated systematic review of randomized controlled trials of carer interventions. Int Psychogeriatr.

[10] Wiegelmann H, Speller S, Verhaert L-M, Schirra-Weirich L, Wolf-Ostermann K (2021). Psychosocial interventions to support the mental health of informal caregivers of persons living with dementia – a systematic literature review. BMC Geriatr.

[11] Cheng ST, Au A, Losada A, Thompson LW, Gallagher-Thompson D (2019). Psychological interventions for dementia caregivers: What we have achieved, what we have learned. Current Psychiatry Reports.

[12] Murfield J, Moyle W, O’Donovan A. Mindfulness- and compassion-based interventions for family carers of older adults: A scoping review. Int J Nurs Stud. 2021:103495 10.1016/j.ijnurstu.2019.103495. 10.1016/j.ijnurstu.2019.103495 31862112

[13] Neff K (2003). Self-compassion: An alternative conceptualization of a healthy attitude toward oneself. Self Identity.

[14] Gilbert P (2014). The origins and nature of compassion focused therapy. Br J Clin Psychol.

[15] Zessin U, Dickhäuser O, Garbade S (2015). The relationship between self-compassion and well-being: A meta-analysis. Applied Psychology: Health and Well-Being.

[16] Kirby JN, Tellegen CL, Steindl SR (2017). A meta-analysis of compassion-based interventions: Current state of knowledge and future directions. Behav Ther.

[17] Lloyd J, Muers J, Patterson T, Marczak M (2019). Self-compassion, coping strategies, and caregiver burden in caregivers of people with dementia. Clin Gerontol.

[18] Murfield J, Moyle W, O’Donovan A, Ware R. The role of self-compassion, dispositional mindfulness, and emotion regulation in the psychological health of family carers of older adults Clin Gerontol. 2020:1–13 10.1080/07317115.2020.1846650. 10.1080/07317115.2020.1846650 33263503

[19] Collins RN, Gilligan LJ, Poz R (2018). The evaluation of a compassion-focused therapy group for couples experiencing a dementia diagnosis. Clin Gerontol.

[20] Gaugler JE, Jutkowitz E, Shippee TP, Brasure M (2017). Consistency of dementia caregiver intervention classification: An evidence-based synthesis. Int Psychogeriatr.

[21] Donkers HW, Van der Veen DJ, Teerenstra S, Vernooij-Dassen MJ, Nijhuis-vander Sanden MWG, Graff MJL (2018). Evaluating the social fitness programme for older people with cognitive problems and their caregivers: Lessons learned from a failed trial. BMC Geriatr.

[22] Slattery P, Saeri AK, Bragge P (2020). Research co-design in health: A rapid overview of reviews. Health Res Policy Syst.

[23] Michie S, Fixsen D, Grimshaw JM, Eccles MP (2009). Specifying and reporting complex behaviour change interventions: The need for a scientific method. Implementation Science.

[24] Morrison L, Muller I, Yardley L, Bradbury K (2018). The person-based approach to planning, optimising, evaluating and implementing behavioural health interventions. The European Health Psychologist.

[25] Yardley L, Ainsworth B, Arden-Close E, Muller I (2015). The person-based approach to enhancing the acceptability and feasibility of interventions. Pilot and Feasibility Studies.

[26] Yardley L, Morrison L, Bradbury K, Muller I (2015). The person-based approach to intervention development: Application to digital health-related behavior change interventions. J Med Internet Res.

[27] Langley J, Wolstenholme D, Cooke J (2018). ‘Collective making’ as knowledge mobilisation: The contribution of participatory design in the co-creation of knowledge in healthcare. BMC Health Serv Res.

[28] National Health and Medical Research Council (2016). Statement on consumer and community involvement in health and medical research.

[29] INVOLVE (2012). Briefing notes for researchers: Involving the public in NHS, public health, and social care research.

[30] Dale J, Loew J, Nanton V, Grason Smith G (2018). Coproduction of a theory-based digital resource for unpaid carers (The Care Companion): Mixed-methods study. JMIR Aging.

[31] Lawrence V, Kimona K, Howard RJ, Serfaty MA, Wetherell JL, Livingston G (2019). Optimising the acceptability and feasibility of acceptance and commitment therapy for treatment-resistant generalised anxiety disorder in older adults. Age Ageing.

[32] Essery R, Pollet S, Smith K, Mowbray A, Slodkowska-Barabasz F, Denison-Day J (2021). Planning and optimising a digital intervention to reduce older adults’ cognitive decline. Pilot and Feasibility Studies.

[33] Craig P, Dieppe P, Macintyre S, Michie S, Nazareth I, Petticrew M (2008). Developing and evaluating complex interventions: The new Medical Research Council guidance. BMJ.

[34] Craig P, Dieppe P, Macintyre S, Michie S, Nazareth I, Petticrew M (2013). Developing and evaluating complex interventions: The new Medical Research Council guidance. Int J Nurs Stud.

[35] Muller I, Santer M, Morrison L, Morton K, Roberts A, Rice C (2019). Combining qualitative research with PPI: Reflections on using the person-based approach for developing behavioural interventions. Research Involvement and Engagement.

[36] Greenhalgh T, Hinton L, Finlay T, Macfarlane A, Fahy N, Clyde B (2019). Frameworks for supporting patient and public involvement in research: Systematic review and co-design pilot. Health Expect.

[37] Bethell J, Commisso E, Rostad HM, Puts M, Babineau J, Grinbergs-Saull A (2018). Patient engagement in research related to dementia: A scoping review. Dementia.

[38] Wang G, Marradi C, Albayrak A, van der Cammen TJM (2019). Co-designing with people with dementia: A scoping review of involving people with dementia in design research. Maturitas.

[39] Goeman DP, Corlis M, Swaffer K, Jenner V, Thompson JF, Renehan E (2019). Partnering with people with dementia and their care partners, aged care service experts, policymakers and academics: A co-design process. Australas J Ageing.

[40] Duncan E, O’Cathain A, Rousseau N, Croot L, Sworn K, Turner KM (2020). Guidance for reporting intervention development studies in health research (GUIDED): An evidence-based consensus study. BMJ Open.

[41] Staniszewska S, Brett J, Simera I, Seers K, Mockford C, Goodlad S (2017). GRIPP2 reporting checklists: Tools to improve reporting of patient and public involvement in research. BMJ.

[42] Murfield J, Moyle W, Jones C, O’Donovan A (2020). Self-compassion, health outcomes, and family carers of older adults: An integrative review. Clin Gerontol.

[43] Murfield J, Moyle W, O’Donovan A (2020). Self-compassion as an applicable intervention target for family carers of older adults: A conceptual commentary. Int J Geriatr Psychiatry.

[44] Murfield J, Moyle W, O’Donovan A. Experiences of compassion among family carers of older adults: Qualitative content analysis of survey free-text comments. Scand J Caring Sci. 2021:1–10 10.1111/scs.13010. 10.1111/scs.13010 34096636

[45] Braun V, Clarke V (2019). Reflecting on reflexive thematic analysis. Qual Res Sport Exerc Health.

[46] Braun V, Clarke V (2006). Using thematic analysis in psychology. Qual Res Psychol.

[47] Braun V, Clarke V (2021). One size fits all? What counts as quality practice in (reflexive) thematic analysis?. Qual Res Psychol.

[48] Braun V, Clarke V (2021). To saturate or not to saturate? Questioning data saturation as a useful concept for thematic analysis and sample-size rationales. Qual Res Sport Exerc Health.

[49] Fox J, Cattani K, Burlingame GM (2021). Compassion focused therapy in a university counseling and psychological services center: A feasibility trial of a new standardized group manual. Psychotherapy Res J Soc Psychother Res.

[50] Carter A, Gilbert P, Kirby JN (2021). Compassion-focused therapy for body weight shame: A mixed methods pilot trial. Clin Psychol Psychother.

[51] Gilbert P (2010). Compassion focused therapy: Distinctive features.

[52] Gilbert P, McEwan K, Matos M, Rivis A (2011). Fears of compassion: Development of three self-report measures. Psychol Psychother.

[53] McLean L, Bambling M, Steindl SR (2021). Perspectives on self-compassion from adult female survivors of sexual abuse and the counselors who work with them. Journal of Interpersonal Violence.

[54] Pauley G, McPherson S (2010). The experience and meaning of compassion and self-compassion for individuals with depression or anxiety. Psychol Psychother.

[55] Gonyea JG, Paris R, de Saxe Zerden L (2008). Adult daughters and aging mothers: The role of guilt in the experience of caregiver burden. Aging Ment Health.

[56] Lacey J, Hiskey S, Andrews L. Compassion focused expressive writing (CFExW) among carers of people with dementia: Some reflections. FPOP Bulletin. 2017;July No.139:32–7

[57] Whitebird RR, Kreitzer MJ, Lewis BA, Hanson LR, Crain AL, Enstad CJ (2011). Recruiting and retaining family caregivers to a randomized controlled trial on mindfulness-based stress reduction. Contemp Clin Trials.

[58] Krouwel M, Jolly K, Greenfield S (2019). Comparing Skype (video calling) and in-person qualitative interview modes in a study of people with irritable bowel syndrome – an exploratory comparative analysis. BMC Med Res Methodol.

